# Research of the Fatigue Life of Welded Joints of High Strength Steel S960 QL Created Using Laser and Electron Beams

**DOI:** 10.3390/ma13112539

**Published:** 2020-06-03

**Authors:** Milan Sága, Mária Blatnická, Miroslav Blatnický, Ján Dižo, Juraj Gerlici

**Affiliations:** 1 Department of Applied Mechanics, Faculty of Mechanical Engineering, University of Žilina, 010 26 Žilina, Slovakia; milan.saga@fstroj.uniza.sk (M.S.); maria.blatnicka@gmail.com (M.B.); 2 Department of Transport and Handling Machines, Faculty of Mechanical Engineering, University of Žilina, 010 26 Žilina, Slovakia; miroslav.blatnicky@fstroj.uniza.sk (M.B.); juraj.gerlici@fstroj.uniza.sk (J.G.)

**Keywords:** mechanical properties, weld joints, fatigue, high strength steel

## Abstract

This study investigated the fatigue life of welded joints, in particular, the welds of the high-strength steel S960 QL. The welds were created using unconventional technologies by utilising laser and electron beams. The direct application of the research is intended to be carried out through implementing the results towards the design of tracks for the track-wheel chassis of the demining system Božena 5. The producer’s experience shows the damage found in the current track design. The damage occurred during reversing the vehicle on a sand surface. Our goal was to solve this problem. The information acquired in this research will be a very important input factor for further designs of the track made of the tested material and its welds. The analysis of the residual stresses was also part of this study. The experimental research of the tested material’s fatigue life and welded joints was realised on the specimens loaded using cyclic bending and cyclic torsion. These loads were dominant during the track operation. The fatigue life of the tested material was detected using a device designed by us. The measurement results were processed in the form of the Wöhler’s S–N curves (alternating stress versus number cycles to failure) and compared with the current regulations issued by the International Institute of Welding (IIW) in the form of the FAT curves (IIW fatigue class). The achieved research results indicate that the modern welding technologies (laser and electron beams) used on the high-strength steel had no principal influence on the fatigue life of the tested material.

## 1. Introduction

Steel seems to be one of the most important materials in engineering practice. The history of steel dates back to more than two millennia ago, and since that time, its production procedures and utilisation properties have been constantly improved. 

One of the main reasons for the broad implementation of steel is the occurrence of iron ore and relatively rapid and low-cost production. A reduced weight can be achieved by using high-strength steels. The replacement of the conventional structural steels with high-strength steels will cause a significant change in the individual members’ cross-section without changing their carrying capacity. This fact naturally leads to savings in the amount of required material [1,2].

**Figure 1:**
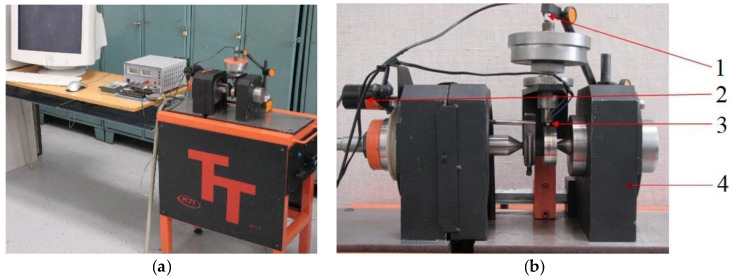
Experimental workplaces for measuring (**a**) the material wear and (**b**) the detail of the testing device: 1—a measuring element TESA, 2—a speedometer, 3—a specimen with a thermocouple and 4—an axlebox.

**Figure 2:**
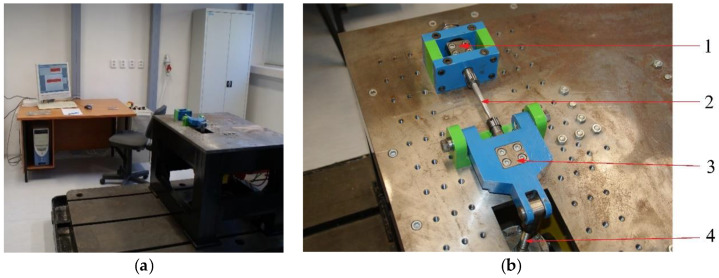
Experimental workplaces for (**a**) the material fatigue and (**b**) the detail of the testing device: 1—a rocker for torque generation, 2—a specimen, 3—a rocker for bending moment generation and 4—a load cell.

This research aimed to investigate the mechanical properties of the high-strength steel S960 Q, mainly after welding. Using this steel instead of structural steel S500 MC would reduce the weight of products and improve the lifecycle in severe operational conditions, though this will incur a higher material cost. As the application is considered for the production of weldments, the process of replacement of the structural steel by high-strength steel is accompanied by extensive research. This research is mainly aimed at the assessment of welding effects on the strength, tribological properties, fatigue life and hardness of the material. Research activities also include the design of unique test devices, i.e., a tribotester for research on the tribological properties and a multiaxial material fatigue device for researching material properties under the multiaxial loads (Figure 1 and Figure 2). This research aimed to show our efforts regarding showing the suitability of the intended usage of the tested material.

However, for technical practice, more than just the extraordinary strength properties of steel is important. There is a tendency to produce machines and equipment from steel with a higher transferred output. Of course, this is valid also for the Božena 5 equipment. In connection with this, there is a requirement to constantly work towards reducing the weight of the used components. This is the reason why we used high-strength steel. It is also desirable to increase the speed of individual members of the mechanisms. The operation temperatures increase or a combined load occurs. In the case of Božena 5, this occurs due to the detonations of the searched mines. The high-strength steel components can bear all of this thanks to the new manufacturing technologies and heat treatments. Lastly, in practice, the components are connected into larger components, which leads to requiring new technological methods of joining the material. The welding processes and the area of weldability using individual methods can be included here. Otherwise, the lifetime of steels could be rapidly reduced [3,4].

The appropriate weldability of steel means not only making the weld to correctly fulfill its function but it is also necessary for the area influenced by the heat to achieve mechanical properties similar to the base material [5]. The steel is appropriately weldable if it does not require any special conditions for making the weld of the required quality. The main parameter determining the weldability of the steels is the carbon content and the carbon equivalent. It can be determined using two methods, namely the method AC.2—the carbon equivalent *CE*(*CEV*) in wt%:

(1) $$CE(CEV)=C+\frac{Mn}{6}+\frac{Cr+Mo+V}{5}+\frac{Ni+Cu}{15},$$

or using the method BC.3—the carbon equivalent *CET* in wt%:

(2) $$CET=C+\frac{Mn+Mo}{10}+\frac{Cr+Cu}{20}+\frac{Ni}{40}.$$

The method AC.2 is used for classically produced steels, while the method BC.3 is used for the micro-alloyed, ultrafine-grain, high-strength steels. The main emphasis is on eliminating the hydrogen (cold) cracking that results in creating the cold cracks [6,7].

A significant problem that limits the usage of high-strength steels is material fatigue [8]. It is a very broad area that began to be solved in the 19th century when a lot of accidents occurred due to damaged railway axles and other railway components [9,10]. Since that time, scientists have been thoroughly investigating this phenomenon to understand its essence and to implement the knowledge into practice [11,12,13]. It seems that as soon as they understand certain interconnections, other areas for research emerge [14,15]. One of these areas is undoubtedly the fatigue of welded joints. According to the conservative recommendations of the International Institute of Welding [16], the fatigue life of welded structures is more or less independent of the material yield point. Based on this statement, we can conclude that the implementation of the high-strength steels does not improve the fatigue life of the structure in the condition without adaptations after welding; we decided to carry out the research presented in this article to verify or reject this statement. Moreover, the bigger the material yield point, the more sensitive its fatigue strength becomes due to the presence of notches and surface conditions of the material. Furthermore, because there are always notches in the welded material connected with a change in the cross-section of the joint being welded, the welds of high-strength steels are said to be prone to material fatigue [4,17].

We hypothesised that better fatigue properties can be achieved by using high-quality welded joints. There are several possibilities regarding how to achieve it: by reducing the surface defects or using the appropriate welding methods [18,19] and also by implementing an additional adaptation of the welds through utilising other methods, e.g., HFMI (high-frequency mechanical impact) or TIG (tungsten inert gas) dressing (melting the weld root). It was proved that the residual stresses in the material affected its fatigue life. In particular, the residual stresses in the material affected the medium stress of the fatigue cycle and it was detected that with the increasing ultimate strength of the steel, the effect of the medium stress on fatigue life also increased [20,21,22].

The usage of the high-strength steels is affected by the fact that the new knowledge about them is processed slowly into the standards and regulations. The designed standards, regulations and general knowledge are based on using steels with a lower strength and utilising conventional welding technologies. There is a clear need to teach engineers designs using high-strength steels and to involve this knowledge into the standards and rules without too much conservatism. The implementation of high-strength steels in the designs is regulated in the European countries by the EU standard Eurocode 3: Design of Steel Structures [23]. Sections 1–12 discuss the usage of steels up to class S700; however, it does not contain a sufficient quantity of experimental data and information about the characteristics and properties of the structural joints in these steels [23]. Currently, experts are working on including additional rules for steels up to S960. This presented part of the whole study concentrates mainly on investigating the fatigue life of the high-strength steel S960QL and its change after implementing the welded joints using the laser and electron beam welding technologies.

The fatigue of material is thus a very complex physical process. Its essence has not been exactly described until now and the fatigue of welds is even more complicated. During the welding process, the material is heated and subsequently cooled. Then, the base material is melted and it can be mixed with filler material. This will cause an increase in the inhomogeneity and a physical and chemical difference, penetration of inclusions, pores, hollows, etc. The lacks of fusion and the shape of the welded joint’s cross-section create conditions for a high concentration of stress. Residual stresses develop, which then affect the fatigue properties of the joint [24].

The fatigue of the welded material affects a wide range of applications, e.g., ships, cars, cranes, bridges, etc. Therefore, several approaches for assessing the fatigue damage of welded joints have been developed. There is a lot of literature written on fatigue tests and the development of approaches used to consider all parameters that affect the fatigue [25,26,27,28,29]. The individual approaches are classified according to what level of the “local character” the stresses or deformations caused by the external load. Then, we could classify the approaches according to the assessment method for strains (deformations) at the global, design and local levels. However, further classification includes a division according to the stress and deformation approach, as well as in terms of fracture mechanics. 

The content of the paper introduces the achieved results in the field of research of the properties of high-strength steel for the production of some highly loaded components of demining equipment. The novelty of the presented research consists of the implementation of the tested high-strength steel S960 QL for the production of track shoes for the demining equipment.

## 2. Tested Material

**Figure 3:**
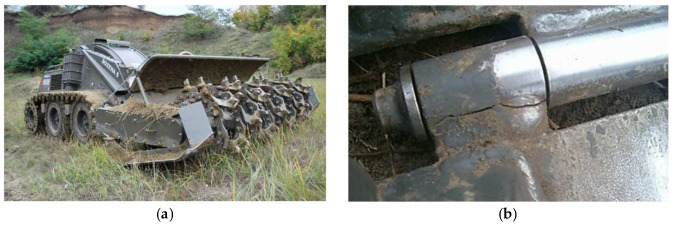
(**a**) Demining equipment Božena 5 and (**b**) an image of the weld damage of the currently used steel track caused by its operation.

A material in its essence is a substance that is used for further processing, where a more complicated product is created by several material components. A material’s usage is limited by both its partial and complex properties. They can carry out relative motion between each other. Suitable connections of common machine components will create a purposeful structure, i.e., a mechanical device is called a mechanism. This is also the case for the demining equipment Božena 5 (Figure 3a). In our case, the track is an exposed component of this vehicle and thus needs to be carefully optimised (Figure 3b). Each part of this working vehicle is a small and closed unit. Its task differs from the tasks of other parts. Each component (produced from the material) has a defined function for its appropriate operation.

From a complex point of view, we determined suitable material for the track (supported by experimental tests), optimised its design (supported by the FEM simulations) and suggested the manufacturing technology (welding). We justified the selection of the tested material in terms of its availability, mechanical properties and excellent weldability. The base material of the test specimens was Strenx 960, which is the name for high-strength steel with a yield point of 960 MPa. The basic dimensions of the weldment were as follows: length of 200 mm, width of 100 mm and thickness of 10 mm. This name covers a group of structural steels (S) that were produced under various designations, e.g., Docol, Optim, Domex and Weldox with various yield points of 700, 900, 960 and 1100, and the latest one with 1300 MPa. Individual steels of the Strenx group are nowadays differentiated by the additional designations CR (cold-rolled structural steel), MC (hot-rolled structural steel) or Plus. The standard EN10025-6 [30] determines the chemical composition of the steel S960QL, as seen in Table 1.

The steel used for the research was Strenx 960 without any additional designation in the form of 10 mm boards, which was designated Weldox 960 E. It is a high-strength structural steel material and the producer guarantees fulfilment of the standard EN 10025-6/S960QL [30]. It is thus hot-roll-treated high-strength structural steel (hardened and tempered) (Q) and maintains its properties at low temperatures (L); the chemical composition of the given steel as provided by the manufacturer (Strenx®960, SSAB, Stockholm, Sweden) is given in Table 2.

Table 3 provides the real chemical composition of the given batch used for the research. It was provided by the company Winfa, Ltd., Trnava, Slovakia. This company was also the supplier of the material for this research. A delivery note was attached to the material and its data is given in Table 3. 

The carbon equivalents were *CEV* = 0.54 wt% and *CET* = 0.359 wt%. These values indicated the need for pre-heating for welding, where the manufacturer recommends preheating to 75 °C for material thicknesses over 10 mm, while up to 10 mm, no preheating is necessary. The mechanical properties detected by the tensile test and the tests of the impact strength given by the manufacturer are shown in Table 4.

**Figure 4:**
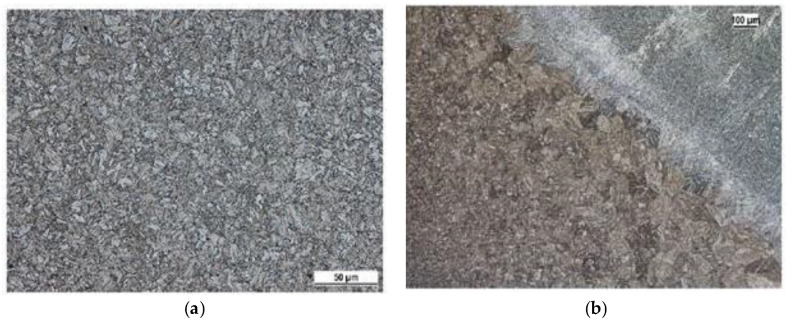
(**a**) Microstructure of the base material Strenx 960 and (**b**) a transfer of the metal being welded to the area affected by the heat using the MAG (metal active gas) technology.

**Figure 5:**
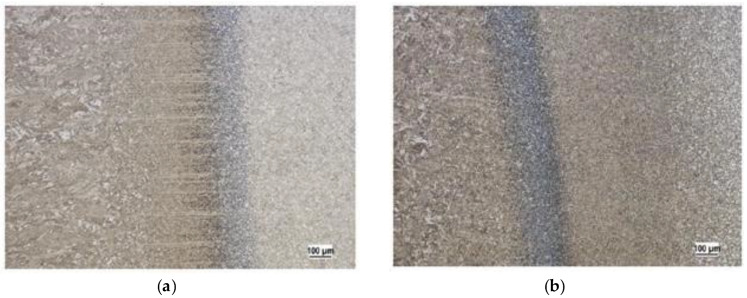
(**a**) A transfer of the metal being welded (its microstructure) to the area affected by the heat of the material Strenx 960 using the electron beam technology and (**b**) a transfer of the metal being welded into the area affected by the heat using the laser beam technology.

The microstructure (Figure 4 and Figure 5) of the base material Strenx 960 and also the change of the structure after welding by individual technologies (laser and electron beam, MAG (metal active gas)) was observed to acquire a more detailed overview regarding the tested material. Figure 4a shows the microstructure of the high-strength steel Strenx 960. It had a fine-grain structure created by the low-carbon tempered martensite. All the specimens were produced using an accurate saw for cutting the metallographic specimens. Ten specimens were used for this research. The specimens were taken from the middle parts of the welded plates. We used a thin cutting wheel cooled by a coolant to avoid affecting the surface with heat. The specimen surface was then cleaned and degreased. Polishing to remove the deformed layers of the material followed. The last step was the etching of the specimens’ surfaces with 2% Nital (a solution of nitric and alcohol; ATM Ltd., Mammelzen, Germany) (2% of HNO_3_ + 98% of ethyl alcohol).

We observed a martensite structure in all the microstructures. This can be explained by the speed of the welding, and in particular, is directly connected with the speed of cooling. The speed of cooling, especially in the case of the laser and electron welding, is usually higher than the critical speed of cooling for creating the martensite structure. We showed that the given progressive high-strength steel S960 was typical in terms of its high hardenability. This is a very useful fact for the considered utilisation of the tested steel. Along with fatigue, the track of the equipment Božena 5 suffers from wear during its operation. The wear is of an adhesive and abrasive character. The higher hardness reached in the process of welding will mean a better abrasive resistance. This is the reason why we are currently working in this interesting area.

## 3. Experimental Welding Process and Assessing Its Influence on the Material Strength

Before undertaking the experimental fatigue tests on the specimens, a range of scientific and research activities was carried out. They were especially connected with the design and production of the test specimens for cyclical stress due to bending and torsion, as well as with calibrating measurements, i.e. adjusting individual load levels for the particular type of load (bending, torsion). Lastly, research was also done to help create a final element model for detecting the state of stress and deformation at the critical point of the fatigue specimen.

The welding of the steel boards was done in collaboration with a certified external partner. Laser and electron welding technologies were our focus. The laser welding was done using the solid-state YLS fibre laser (First Welding Company, Inc., Bratislava, Slovakia) with an output of 5 kW, a wavelength of 1.06 μm and a Precitec YW52 laser welding head with the optical fibre diameter of 0.1 mm. The material was clamped without the root gap and the tack welds were used at its ends. The electron beam welding was done using a PZ EZ JS30 JUMBO welding system (First Welding Company, Inc., Bratislava, Slovakia) with an output pulse regulation from 0 to 8 kW and a beam voltage from 30 to 60 kV. The material was clamped without the gap and the tack welds were used at its ends. The welding operation was done in a continuous regime. For both technologies, the welding surfaces were fine-milled and defatted using acetone to ensure the material’s cleanliness and weld quality before welding. The individual welding parameters are shown in Table 5 and Table 6.

Based on the aforementioned data and Equation (3), we calculated the welding heat input for individual methods. As we can see in Table 7, the quantity of heat brought to the material during welding per unit of length is larger for the laser beam welding than electron welding:

(3) $$Q=k\times \frac{I\times U}{1000\times \nu }=k\times \frac{P}{1000\times \nu }$$

where *k* is the thermal efficiency coefficient of the welding process for both the laser and electron beam welding k_ray_ = 0.95.

**Figure 6:**
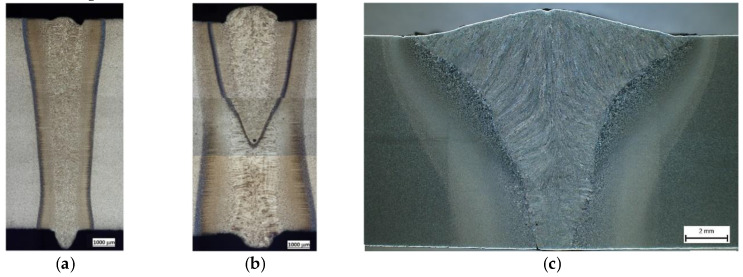
Microstructures of the welded joints of the tested material S960 QL: (**a**) electron weld, (**b**) laser weld and (**c**) the weld using the MAG technology.

As the middle of Figure 6a shows, the weld using the electron beam had a smaller width than the laser beam weld. However, both technologies ensured a visibly narrower heat-affected zone (HAZ), along with the weld itself, than in the case of the MAG technology (Figure 6c). This figure also shows that the weld was created in two steps. First of all, the basic layer was placed and the covering weld bead was placed on top. The whole welded joint, as well as the HAZ, was considerably wider compared with the previous technologies. Currently, only the MAG technology is utilised for producing the track of the Božena 5 equipment. We aimed to replace this technology with other, more progressive methods, such as laser and electron welding). The reasons are clear based on the previous and next results of this research.

**Figure 7:**
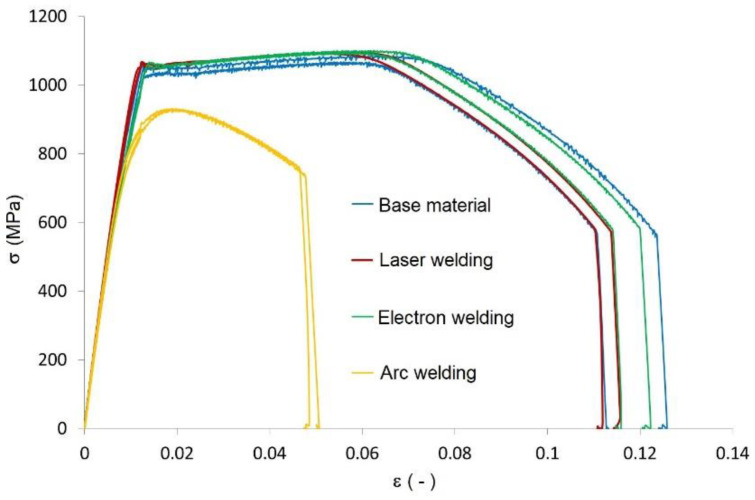
Documentation diagram of comparing the tensile diagrams of the base material and the welded ones.

Due to the subsequent experimental and numerical testing, first, the static tensile test (Figure 7) was carried out. It was done on a hydraulic universal testing equipment INOVA (INOVA Ltd., Bad Schwalbach, Germany) in our lab according to the standard ISO 6892 [33]. The tensile tests showed that the welds created by the laser or electron beam did not affect the material’s strength. The specimens were broken (10 test specimens were tested for the base material and each type of weld) welded outside the weld, i.e., in the base material, for both laser-welded and electron-welded material. The values of the yield point, as well as of the ultimate strength, exceeded the minimum values for the base material defined by the supplier. In all three cases (the base material, and welded by the laser or electron beam), the material showed a lower elongation percentage than that given by the producer. The average elongation was 11.32% based on ten measurements, while the manufacturer gave a value of 15%.

Along with comparing the investigated technologies (laser and electron welding) with the base material in Figure 7, we also show the results of implementing an arc weld, where the specimens were broken in the place of the weld; the ultimate strength did not reach the minimum value of the yield point of the base material (Table 8).

There was a drop in the yield point of approximately 20% and of the strength by approximately 15%. Furthermore, the ratio of the yield point and the ultimate strength changed from 0.96 to 0.88. Moreover, the material also showed a very low ductility of 4.3% on average (Table 9).

In this section, we summarise the procedures utilised for creating the welds on the tested material, specifically, the welding parameters and differences in the heat inputs of individual technologies are presented. These partial results indicate a connection between the heat input and weld width of the heat-affected zone. The experimental tensile test showed the minimal influence of the progressive welding technologies on the material’s strength. In contrast, the arc welding process showed a decrease in strength. Furthermore, these findings will help achieve the optimisation of the vehicle track solution. The residual strength, and especially the yield strength, will be a suitable tool for designing according to this important factor. However, this problem is complex and difficult to solve. Therefore, it is extraordinarily important to create an analysis of the residual stresses from welding. They represent one of the limiting factors of a design’s fatigue life.

## 4. Analysis of Residual Stresses

Residual stress is defined as stress that occurs in a connected body when no external load affects it. Heating, melting and cooling of the weld and the non-uniform distribution of heat cause plastic thermal deformation. This results in permanent deformation and the development of residual stresses near the weld and the heat-affected zone. The tensile residual stresses caused by welding usually have a negative influence on the fatigue life of the structures, resistance against corrosion and other chemical properties. On the other hand, the pressure residual stresses improve the fatigue life (strength) of the material. Regarding the fatigue life, it is extraordinarily important to investigate the distribution of the residual stresses after welding [34,35,36].

The residual stress calculation requires implementing a lot of simplifications due to the number of variables affecting the result. These assumptions enable a mathematical description of the development but they also simplify its physical essence. However, the calculations of the residual stresses remain complicated. Therefore, despite the extensive progress in the area of the computer techniques and good results of the simulation processes, residual stresses are very frequently detected by implementing the experimental methods [37].

The residual stresses are traditionally measured using destructive and non-destructive methods. The boring and separation methods are examples of destructive methods and the X-ray diffraction method is an example of a non-destructive method [38,39,40,41].

The measurement of the residual stresses was undertaken on an X-ray Proto XRD diffractometer (Proto Manufacturing Inc., Taylor, MI, USA). Using the X-ray diffraction method, we measured the deformation in the crystal lattice due to residual stresses. The residual stresses connected with it were determined from the elastic constants with an assumption of linear elastic deformation at the corresponding level of the crystal lattice.

**Figure 8:**
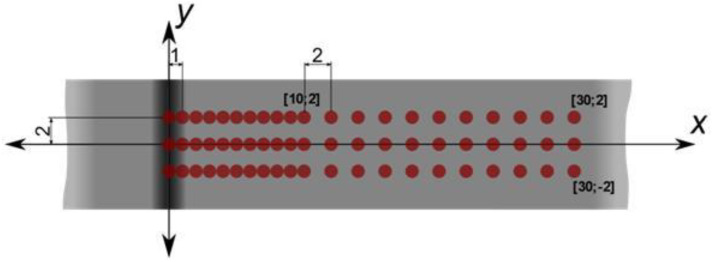
Schematic depictions of the measured points on the test specimen’s surface.

The measurement was carried out in two directions on the test specimen surface (in the direction parallel with the weld and perpendicularly to the weld) at the points shown in Figure 8. A coordinate matrix of all points was created, in which the stress status in one direction was measured in an automated way. This means that two measurement series were produced.

**Figure 9:**
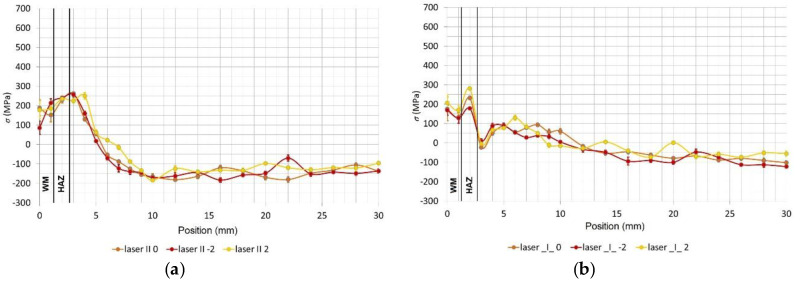
(**a**) The distribution of the longitudinal residual stresses of the test specimen surface welded using the laser beam as a function of the distance from the weld, measured in three lines and (**b**) the distribution of the transverse residual stresses of the test specimen surface welded using the laser beam as a function of the distance from the weld, measured in three lines; WM—weld metal, HAZ—heat affected zone.

**Figure 10:**
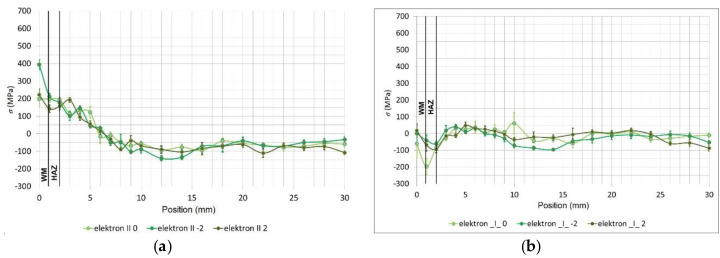
(**a**) The distribution of the longitudinal residual stresses of the test specimen surface welded using the electron beam as a function of the distance from the weld, measured in three lines and (**b**) the distribution of the transverse residual stresses of the test specimen surface welded using the electron beam as a function of the distance from the weld, measured in three lines.

Figure 9 and Figure 10 give individual measurements of the residual stresses. The stresses were measured in three lines on the test specimen surface and their distance was 2 mm from each other. The measurements for both welding technologies were carried out on the surface side of the weld in the central area of the welded board where the welding parameters were stable to avoid any distortions of the results. In the framework for the first 10 mm, the gap between individual points was 1 mm. In the next part, the gap was enlarged to 2 mm. The total distance from the middle of the weld, up to where the measurement was taken, amounted to 30 mm.

In Figure 9a, we can see the distribution of the longitudinal residual stresses (in the direction parallel to the weld) on the surface of the laser-welded specimen with the marked regions of the welded metal and the heat-affected zone (HAZ). The tensile residual stresses in the weld were 200 MPa, and the maximum value of 250 MPa was found behind the HAZ at a distance of 3 to 4 mm; in this region, the stresses decrease to a maximum negative pressure of −200 MPa. The residual stresses in the cross direction for the laser weld are in Figure 9b. Directly in the weld, 200 MPa of tensile strength was found; this increased to 280 MPa in the HAZ, and from this point, the tensile strength decreased until a constant pressure of −100 MPa was found

The distribution of the longitudinal residual stresses in the test specimen welded using the electron beam is in Figure 10a. The maximum value in the weld was ambiguous: in two lines, a value of 215 MPa was found, but in the third, it increased up to 400 MPa. From this point, it gradually dropped and remained constant at a value of approximately −100 MPa. Regarding the transverse residual stresses in the specimen welded using the electron beam (Figure 10b), directly in the weld, moderate pressure values of −50 MPa were found, and from this point, they continued decreasing until the end of HAZ, where they moderately increased to the maximum tensile strength of 50 MPa and finally stabilised at a pressure of 0 to −25 MPa.

The comparison of the average values of the longitudinal residual stresses on the test specimen surface welded using different technologies shows that the implemented technologies caused the development of the tensile stresses in the weld. The lowest values of the stresses were achieved for the laser weld, although a peak was recorded between the HAZ and the base material, which could represent a potential risk area [42,43,44]. However, the difference between the stresses for the laser and electron beam welding was minimal.

The comparison of the average values of the transverse residual stresses seems to be more interesting. There were considerable differences in the stress values for these individual technologies. In the case of the laser weld, an average value of up to only 250 MPa was found, and for the electron weld, even a negative value was found, i.e., the residual stress with a pressure of −100 MPa. The connection between the thermal input during welding and the transversal residual stress after welding was clearly confirmed here. Currently, we are working on creating such an analysis the MAG technology as well. We hope it will bring verification and more accurate information about the aforementioned statement.

## 5. Investigating the Fatigue Life of the Base and Welded Material S960

The experimental research of the fatigue curves on the tested specimens was done on our fatigue-testing equipment. The testing of the specimens was carried out by implementing the so-called controlled deformation amplitude approach. The controlled parameter for the torsion load was the amplitude of the shearing deformation, i.e., chamfer *γ_xy_*. The controlled parameter for the bending load was the amplitude of the proportional deformation *ε_xx_*. The mechanism of the testing device for the tension load had a design with an eccentric pair with an eccentricity of 4 mm, and for the bend, a pair with eccentricities of 2 mm was used. The selection of these eccentric pairs was done based on the FEM analysis. It was found that the extent of the achieved stresses on the individual loading levels for the given eccentric pairs corresponded best with the material strength detected during the static tests.

The process of loading was carried out with a constant deformation amplitude, a zero mean value, a symmetric (asymmetric coefficient of the cycle *R* = −1) sine cycle, and a loading frequency of 35 Hz. The temperature in the lab was in the range of 20 ± 3 °C. The recorded numbers of the cycles corresponded with the lifespan of individual specimens up to the moment of fracture; in the case of the lifespans higher than 10^7^ cycles, the tests were interrupted.

**Figure 11:**
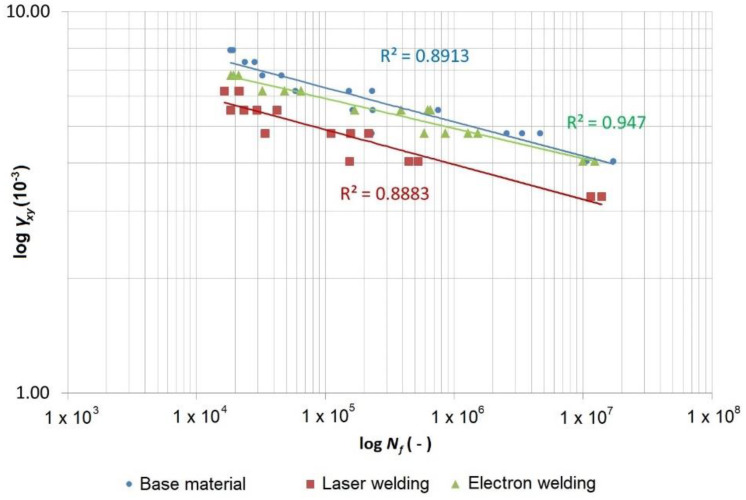
Manson–Coffin curves of the material Strenx 960 and its laser and electron welds loaded using cyclic torsion.

**Figure 12:**
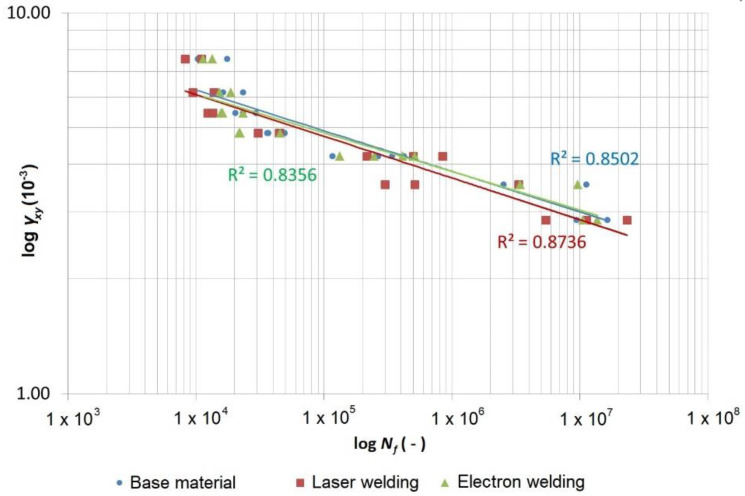
Manson–Coffin curves of the material Strenx 960 and its laser and electron welds loaded using cyclic bending.

The records of the number of cycles for individual materials/technologies and the loading methods were processed in the form of the Manson–Coffin curves (Figure 11 and Figure 12), with the introduced determination coefficients *R*^2^ of the power regression model fitted to the data.

**Figure 13:**
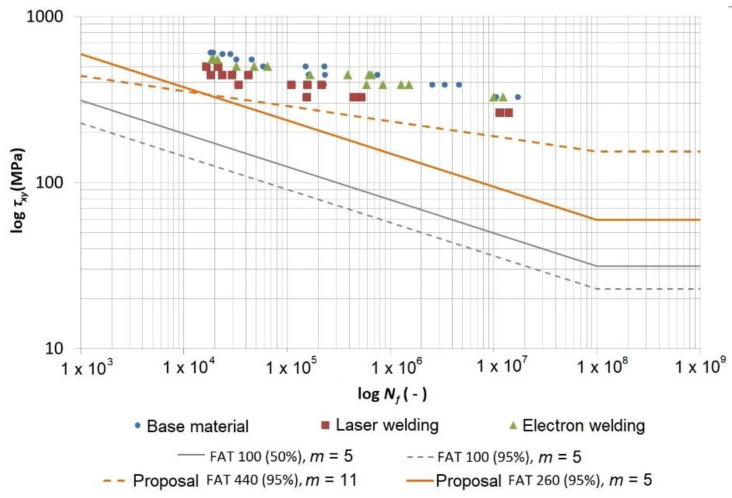
Comparing the data measured with FAT curves recommended by the International Institute of Welding (IIW) for the shear stress in the weld and a proposal of new FAT curves.

**Figure 14:**
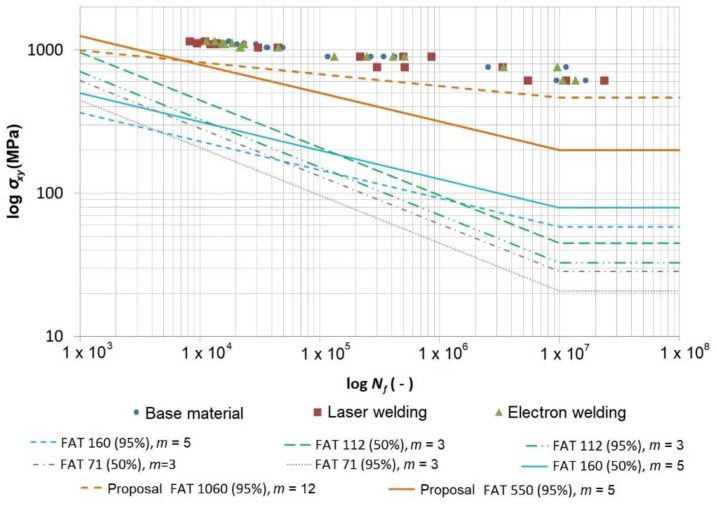
Comparing the data measured with FAT curves recommended by the IIW for the direct stress in the weld and a proposal of new FAT curves.

The data was also expressed in the form of the shear stress *τ_xy_* and the direct stress *σ_xx_* as a function of the number of cycles to fracture (Figure 13 and Figure 14), where the FAT curves (IIW fatigue class), recommended by the International Institute of Welding (IIW), are also depicted. The FAT curves recommended by the IIW are in the form of Wöhler’s S–N curves (alternating stress versus number cycles to failure), for which the following equations are valid:

(4) $$N=\frac{C}{\Delta \sigma^{m}}\;\mathrm{and}\;N=\frac{C}{\Delta \tau^{m}}$$

where *m* is the inclination of the curve that achieves the value of *m* = 3 (if not introduced differently) for the steels loaded using the direct stress up to 10^7^ cycles, and *m* = 5 for the steels loaded using the shear stress up to 10^8^ cycles.

The value or the numerical designation of the FAT curve represents a value of the stress range (Δ*σ* or Δ*τ*) in MPa at the number of cycles *N* = 2 × 10^6^. This means that, e.g., FAT 100 for the shear stress with a determined inclination of the curve *m* = 5 will have a range of the shear stress Δ*τ* = 100 MPa at the number of cycles *N* = 2 × 10^6^ such that the constant *C* will be *C* = *N*·Δ*τ =* (2 × 10^6^)(100) = 2 × 10^8^. With the known value of the constant, it is then possible to calculate the value of the shear stress range using an arbitrary required number of cycles, or vice versa, from Equation (4). The nominal value of the stress range should not exceed 1.5 times the yield point for the direct stress and 1.5 times *R_e_*/√3 for the shear stress.

The highest FAT curve allowed by the IIW for the direct stress is FAT 160 with an inclination of *m* = 5. This curve is to be used for the non-welded parts of the structure. A necessary condition of the usage is the grinding of sharp edges and surface errors. A higher (less specified) FAT curve can be used for the high-strength steels if it is verified by testing. The highest allowed curve for the welded joints is FAT 112 with an inclination of *m* = 3. It is valid for a transversely loaded weld in an X or V shape (welding from both sides) and grinding the overlap and foot of the weld at 100% of the NDT inspection (non-destructive testing methods). The permissible curve FAT 71 is for a transversely loaded butt weld that is welded from one side without backing with the weld root checked using NDT.

The IIW gives only two curves for the welds loaded by the shear stress. FAT 100 is to be used for the base material or the fully penetrated butt weld, while FAT 80 is used for a partially penetrated butt weld or a fillet weld. The FAT curves recommended by the IIW represent the characteristic values calculated from the mean values with a 95% probability of survival (5% probability of failure) based on the bilateral 75% reliability interval. However, the fatigue curves in the diagrams are developed through a regression of the measured data with a 50% probability of survival (50% probability of failure). Therefore, for comparing the measured data with FAT curves, it is necessary to re-calculate *FAT*_95%_ to *FAT*_50%_ using Equation (5) [27]:

(5) $$FAT_{50\%}=\frac{FAT_{95\%}}{10^{-2\times 0.0688}}\approx \frac{FAT_{95\%}}{0.728}$$

The approximate value of the coefficient for recalculating the difference between the mean value and the characteristic curve calculated based on the IIW results is 1.37.

Figure 13 depicts the measured data from the cyclic tests for the torsion-tested specimens. The diagram is in the logarithmic coordinates of the shear stress amplitude versus the number of cycles to fracture. This data shows (similar to the Manson–Coffin curves in Figure 10) that the base material achieved the highest curve, then the material welded using the electron beam was a little bit lower and the lowest curve belonged to the material welded using the laser beam. An important fact is that both curves belonging to the welded specimens showed a very small decrease compared with the base material. It is possible to say that all measured points were high above the curve recommended by the IIW FAT 100 curve, even in the case where it was recalculated on a curve with a 50% probability of survival.

Through the data acquired by testing the specimens welded using a laser, we fitted the power regression model curve to the data using the least-squares method, whose equation can be expressed in the following form, according to Basqin (6):

(6) $$\tau_{a}=1133.3\times N_{f}{}^{-0.091}\;\mathrm{or}\;\tau_{a}=566.65\times {\left(2\times N_{f}\right)}^{-0.091}$$

This equation was rearranged into a power form that complies with the FAT curves, as follows:

(7) $$N=\frac{1133.3^{\frac{1}{0.091}}}{\tau_{a}^{\frac{1}{0.091}}}\approx \frac{1133.3^{11}}{\tau_{a}^{11}}$$

where a cycle number of 2 × 10^6^ gives *τ_a_* ≈ 303 MPa, and thus Δ*τ* ≈ 606 MPa. The 50% FAT 606 with an inclination of *m* = 11 would correspond with this curve and this complies with the 95% FAT 440 with an inclination of *m* = 11 according to Equation (5). However, the IIW determines the inclination of the curve to be *m* = 5, and therefore, if we wanted to maintain this inclination, the FAT 260 (95%) with an inclination of *m* = 5 would correspond with this requirement.

Figure 14 depicts the data measured during the cyclic bending tests for the tested specimens. The diagram is in the logarithmic coordinates of the direct stress amplitude versus the number of cycles to fracture. This data shows (similar to the Manson–Coffin curves in Figure 12) that the base material produced the highest curve. If the number of the cycles was lower (10^4^), the curves of the material welded using a laser or electrons were approximately at the same level but slightly under the curve of the base material. As the number of the cycles increased, the curve for the material welded using electrons increased and at 10^7^ cycles, it equalled the base material. However, in general, the difference between the measured values of the base material and the welded materials was very small. All measured points were safely over the highest curve recommended by the IIW for the direct stress, which was FAT 160 with an inclination of *m* = 5. This was valid also in the case of the 50% version, although this curve was determined for the non-welded parts of the material.

Similarly, as in the case of the data for the cyclic load by torsion, through the data for the cyclic load by bending (laser), we also fitted power regression curves whose equation, according to Basqin, is as follows:

(8) $$\sigma_{a}=2439.8\times N_{f}{}^{-0.083}\;\mathrm{or}\;\sigma_{a}=1219.9\times {\left(2\times N_{f}\right)}^{-0.083}$$

which was rearranged into the power form as follows:

(9) $$N=\frac{2439.8^{\frac{1}{0.083}}}{\sigma_{a}^{\frac{1}{0.083}}}\approx \frac{2429.8^{12}}{\sigma_{a}^{12}}$$

where a cycle number of 2 × 10^6^ gives *σ_a_* ≈ 728 MPa, and thus, Δ*σ* ≈ 1456 MPa. The 50% FAT 1456 with an inclination of *m* = 12 would correspond with this curve and this complies with the 95% FAT 1060 with an inclination of *m* = 12 according to Equation (5). However, the IIW determines the inclination of the curve to be *m* = 5, and therefore if we wanted to maintain this inclination, the FAT 550 (95%) with an inclination of *m* = 5 would correspond with this requirement (Figure 14).

The equations of the Wohler and Manson–Coffin curves recommended by the IIW for all measured data are in Table 10 and Table 11.

## 6. Assessment

We have presented part of our scientific activities leading to a clear goal. The whole research was created to optimise the material component of the working vehicle Božena 5. This is a piece of modern demining equipment made in Slovakia. The track of this vehicle is an exposed component. Therefore, there are extensive requirements regarding the materials it is made of. In this article, we present:

- The selection of the tested material and its chemical composition as defined by the corresponding standard (EN 10 025-6), the producer (SSAB) and the supplier (Winfa).
- The analyses of the microstructure of the base and welded material for the laser, electron beam and MAG technologies.
- The heat inputs of the individual welding technologies. We explain the influence of the heat input on the weld width. The weld widths are shown in the images of the microstructure.

In further research in this area, we will concentrate on more detailed images of the microstructure to detect errors in the welds, which play an important role in the premature track damage (Figure 3b). 

We also carried out tensile tests. Furthermore, on the basis of their results, we are increasingly certain that the utilisation of the high-strength steel will be an ideal material solution for this given area. No principal influence of the progressive methods (laser or electron beam) on the material tensile strength was found. In contrast, this influence was confirmed in the case of conventional MAG technology, where only this technology is currently used for producing the track. Through achieving a high residual strength, we aim to reach a more suitable track design. This fact will be appreciated by the workers who handle the track segments during assembly.

An important part of this article was the analysis of the residual stresses from welding. The analysis indicated that the residual stresses were significantly large. However, in comparison with the material strength, their values were not high enough for us to consider any replacement of the material. It is necessary to take into account that only longitudinal residual stresses were investigated. We intend to continue the research to understand the transverse stresses, as well as both groups of stresses for MAG technology. 

Maybe the most important part was the investigation of the fatigue life of the tested material. This was a comparison of the welded and non-welded material. In this case, we showed that neither progressive method had a principal influence on the fatigue life of the high-strength steel. Therefore, the fatigue life of the welds was comparable with the base material. This fact will be taken into account in the dimensional calculations for the optimised track design. The designed structural unit will be an ideal solution. However, all of this will be confirmed by subsequent extensive tests of the given transport vehicle.

## 7. Conclusions

The goal of this article was to investigate some mechanical properties of the high-strength steel S960 QL and its welded joints. The focus was a series of fatigue tests on the specimens from the base material as well as material welded using electron and laser beam technologies; these were loaded using cyclic bending and cyclic torsion on a fatigue testing device built in our laboratory. We carried out a comparison of the results for the base material welded using the electron and laser beam technologies.

The fatigue tests showed that the current recommendations of the IIW are very conservative. The currently recommended curve for a welded joint loaded using normal stress that corresponds with the welded joints tested in this work (laser and electron welding) was FAT 71. The highest recommended curve that was primarily determined for the construction areas without welds was FAT 160. However, our research shows that all measured points (for both the base material and the welded material) were well above these curves and it was even possible to use the curve FAT 550. Regarding the welded joints loaded by the shear stress, the IIW recommends only two curves, and for the tested specimens, FAT 100 is suitable. Furthermore, in this case, it was shown that all the measured data was safely above this curve and it was even possible to utilise the FAT 260 curve.

Due to the aforementioned reasons, it is therefore necessary to say that for economical utilisation of the material, we need to consider the economical aspects based on the sources. The research indicated that our experimentally created fatigue curve was therefore the only logical alternative for designing the unit being solved.

## Figures and Tables

**Table 1:** Chemical composition of the steel S960QL according to the standard EN 10 025-6 [30].

Chemical Composition of the Steel S960QL (Max. wt %)														
C	Si	Mn	B	Nb	Cr	V	Cu	Ti	Al	Mo	Ni	N	P	S
0.02	0.80	1.70	0.005	0.06	1.50	0.12	0.50	0.05	-	0.70	2.00	0.015	0.20	0.10

**Table 2:** Chemical composition of the steel Strenx 960 according to the manufacturer SSAB [31].

Chemical Composition of the Steel Strenx 960 (Max. wt %)														
C	Si	Mn	B	Nb	Cr	V	Cu	Ti	Al	Mo	Ni	N	P	S
0.20	0.50	1.60	0.005	-	0.80	-	0.30	-	-	0.70	2.00	-	0.02	0.01

**Table 3:** Real chemical composition of the given batch of steel Strenx 960 [32].

Chemical Composition of the Steel Strenx 960 (Max. wt %)														
C	Si	Mn	B	Nb	Cr	V	Cu	Ti	Al	Mo	Ni	N	P	S
0.16	0.21	1.24	0.001	0.015	0.20	-	0.01	0.004	0.06	0.602	0.06	0.003	0.01	0.001

**Table 4:** Selected mechanical properties of the given batch of steel Strenx 960 as declared by the manufacturer [32].

Offset Yield Strength *R_p_*_0.2_ (MPa)	Ultimate Strength *R_m_* (MPa)	Elongation Percentage *A* (%)	Young’s Modulus *E* (J) at *T* = −40 °C
1009.0	1055.0	15.0	61.0

**Table 5:** Welding parameters for laser beam welding.

Laser Beam Welding			
Output *P* (kW)	Velocity *v* (mm·s^−1^)	Focus Position (mm)	Fibre Diameter (mm)
5000	7.5	−4	0.1

**Table 6:** Welding parameters for electron beam welding.

Electron Beam Welding				
Welding Current *I* (mA)	Beam Voltage *U* (kV)	Velocity *v* (mm·s^−1^)	Focusing (mm)	Vacuum Pressure (Pa)
115	55	11	0	0.09

**Table 7:** Determined values of the thermal inputs for individual welding methods.

	Weld Type	
Thermal input	Electron	Laser
*Q* (kJ·mm^−1^)	0.63	0.55

**Table 8:** Selected mechanical properties of the steel Strenx 960 found using the tensile test.

Material Specimens Strenx 960	Offset Yield Strength *R_p_*_0.2_ (MPa)	Ultimate Strength *R_m_* (MPa)	Elongation *A* (%)
Base material	1026.4	1067.6	11.15
Base material	1044.5	1087.9	12.10
Laser welding	1048.7	1094.9	11.17
Laser welding	1058.3	1091,7	11,14
Electron welding	1063.3	1099.9	11.45
Electron welding	1052.4	1094.7	10.88
Arc welding	835.7	931.5	4.70
Arc welding	811.2	930.2	3.89

**Table 9:** Average values of selected mechanical properties of the steel Strenx 960 and its welded joints obtained from the tensile tests.

Material Specimens Strenx 960	Offset Yield Strength *R_p_*_0.2_ (MPa)	Ultimate Strength *R_m_* (MPa)	Elongation *A* (%)	*R_p_*_0.2_/*R_m_* (-)
Basic, electron welding, laser welding	1049	1089	11.3	0.96
Arc welding	823	931	4.3	0.88

**Table 10:** Equations of the Wohler and Manson–Coffin curves in the power form according to Basqin for the test specimens of the base material Strenx 960 and test specimens welded using laser and electron beams loaded using cyclic torsion.

	Strenx 960—Cyclic Torsion		
Compared material	Shear stress *τ_a_* (MPa)	Shear strain *γ_a_* (-)	Coefficient of regression model determination *R^2^* (-)
Base material	$\tau_{a}=1363.6\times N_{f}^{-0.086}$	$\gamma_{a}=17.694\times 10^{-3}\times N_{f}^{-0.09}$	0.8913
Electron weld	$\tau_{a}=1182.6\times N_{f}^{-0.079}$	$\gamma_{a}=14.640\times 10^{-3}\times N_{f}^{-0.079}$	0.9470
Laser weld	$\tau_{a}=1133.3\times N_{f}^{-0.091}$	$\gamma_{a}=14.029\times 10^{-3}\times N_{f}^{-0.091}$	0.8883

**Table 11:** Equations of the Wohler and Manson–Coffin curves in the power form according to Basqin for the test specimens of the base material Strenx 960 and test specimens welded using laser and electron beams loaded using cyclic bends.

	Strenx 960—Cyclic Bend		
Compared material	Shear stress *τ_a_* (MPa)	Shear strain *γ_a_* (-)	Coefficient of regression model determination *R^2^* (-)
Base material	$\sigma_{a}=2454.1\times N_{f}^{-0.08}$	$\varepsilon_{a}=16.696\times 10^{-3}\times N_{f}^{-0.106}$	0.8502
Electron weld	$\sigma_{a}=2337.4\times N_{f}^{-0.077}$	$\varepsilon_{a}=15.524\times 10^{-3}\times N_{f}^{-0.101}$	0.8356
Laser weld	$\sigma_{a}=2439.8\times N_{f}^{-0.083}$	$\varepsilon_{a}=16.743\times 10^{-3}\times N_{f}^{-0.11}$	0.8736
